# Altered Proteomic Profile of Adipose Tissue-Derived Mesenchymal Stem Cell Exosomes from Cats with Severe Chronic Gingivostomatitis

**DOI:** 10.3390/ani11082466

**Published:** 2021-08-23

**Authors:** Antonio J. Villatoro, María del Carmen Martín-Astorga, Cristina Alcoholado, Casimiro Cárdenas, Fernando Fariñas, José Becerra, Rick Visser

**Affiliations:** 1 Laboratory of Bioengineering and Tissue Regeneration, Department of Cell Biology, Genetics and Physiology, University of Málaga, IBIMA, 29071 Málaga, Spain; ajvillatoro@immunestem.com (A.J.V.); mcmartinastorga@gmail.com (M.d.C.M.-A.); alcoholado.c@gmail.com (C.A.); becerra@uma.es (J.B.); 2 Instituto de Inmunología Clínica y Terapia Celular (IMMUNESTEM), 29071 Málaga, Spain; 3 Research Support Central Services (SCAI) of the University of Málaga, 29071 Málaga, Spain; ccg@uma.es; 4 Grupo Ynmun, Spanish Association for the Research in Immunological and Infectious Diseases, 29071 Málaga, Spain; farinas.inmunologia@gmail.com; 5 Biomedical Research Networking Center in Bioengineering, Biomaterials, and Nanomedicine (CIBER-BBN), 28029 Madrid, Spain; 6 Andalusian Centre for Nanomedicine and Biotechnology-University of Málaga (BIONAND), 29590 Málaga, Spain

**Keywords:** feline, feline chronic gingivostomatitis, mesenchymal stem cells, exosomes, ultra-high-performance liquid chromatography high-resolution mass spectrometry (UHPLC–HRMS)

## Abstract

**Simple Summary:**

Feline chronic gingivostomatitis (FCGS) is a common pathology in cats, related to an aberrant immune response. The cause of FCGS remains elusive, despite extensive investigations. A multitude of conditions and infectious agents have been related, without proof of causation, as follows: virus, bacteria, environmental stress, hypersensitivity, etc. In recent years, therapies based on feline adipose-derived mesenchymal stem cells (fAd-MSC) have become an interesting alternative for the treatment of different complex pathologies in cats. Mesenchymal stem cells secrete a wide variety of therapeutic elements, such as bioactive molecules and extracellular vesicles, such as exosomes. It is essential to characterize these elements, to better understand their mechanisms of action. In this study, we show, for the first time, that the proteomic profile of fAd-MSC-derived exosomes, from calicivirus-positive patients with severe FCGS, is altered. Using bioinformatic tools, we have demonstrated the existence of different proteins in the exosomes from diseased patients, responsible for an altered biological effect. In addition, the exosomes do not only experience changes in their cargo, but are also produced in larger quantities. This study might contribute to the better prediction of the clinical outcomes of mesenchymal stem cell treatments in veterinary patients with immune-mediated diseases, such as FCGS.

**Abstract:**

Feline chronic gingivostomatitis (FCGS) is a pathology with a complicated therapeutic approach and with a prevalence between 0.7 and 12%. Although the etiology of the disease is diverse, feline calicivirus infection is known to be a predisposing factor. To date, the available treatment helps in controlling the disease, but cannot always provide a cure, which leads to a high percentage of refractory animals. Mesenchymal stem cells (MSCs) play a pivotal role in the homeostasis and reparation of different tissues and have the ability to modulate the immune system responses. This ability is, in part, due to the capacity of exosomes to play a part in intercellular cell communication. However, the precise role of MSC-derived exosomes and their alterations in immunocompromised pathologies remains unknown, especially in veterinary patients. The goal of this work was to analyze the proteomic profile of feline adipose tissue-derived MSCs (fAd-MSCs) from calicivirus-positive FCGS patients, and to detect possible modifications of the exosomal cargo, to gain better knowledge of the disease’s etiopathogenesis. Using high-resolution mass spectrometry and functional enrichment analysis with Gene Ontology, exosomes isolated from the fAd-MSCs of five healthy cats and five calicivirus-positive FCGS patients, were pooled and compared. The results showed that the fAd-MSCs from cats suffering from FCGS not only had a higher exosome production, but also their exosomes showed significant alterations in their proteomic profile. Eight proteins were exclusively found in the exosomes from the FCGS group, and five proteins could only be found in the exosomes from the healthy cats. When comparing the exosomal cargo between the two groups, significant upregulation of 17 and downregulation of 13 proteins were detected in the FCGS group compared to the control group. These findings shed light on new perspectives on the roles of MSCs and their relation to this disease, which may help in identifying new therapeutic targets and selecting specific biomarkers.

## 1. Introduction

Feline chronic gingivostomatitis (FCGS) is an oral mucosal inflammatory pathology, with an estimated prevalence between 0.7 and 12% in the cat population, and a complicated therapeutic approach [1]. Although its etiopathogenesis is still poorly known, it includes an important immune-mediated component that is associated to multiple factors. Among these, feline calicivirus infection is one of the main predisposing factors of the disease [2,3,4,5,6]. The current treatment, which includes exodontia and the use of different immunosuppressant drugs, helps in controlling the disease, but does not provide a cure in around 30% of the cases. This leads to a high percentage of the patients becoming refractory [7,8].

Mesenchymal stem cells (MSCs) are known to play an important role in the homeostasis and repair of numerous tissues, as well as the ability to modulate the responses of both the innate and the adaptative immune system [9,10]. As to date, there is a high consensus on the fact that their mechanism of action is mainly paracrine, involving the release of a variety of elements that are jointly known as their secretome [11,12]. Among the different components of the secretome are a group of extracellular vesicles, known as exosomes, which have been shown to display high immune-regulating and regenerative properties. Exosomes are nanovesicles originated through the endocytic pathway, with a size varying between 40 and 100 nm, capable of transporting different types of small molecules (cargo) that include nucleic acids, proteins, and lipids, among others. The exosome cargo is involved in communication between cell lineages and has a pleiotropic effect [13,14]. Our group was the first to describe the exosomes derived from feline adipose tissue-derived MSCs (fAd-MSC), demonstrating that they play a role in several biological functions [15].

Recently, the exosomes have become particularly interesting, because of their potential diagnostic and therapeutic applications [16]. On one hand, they have shown high safety and efficacy as therapeutic agents in different disease models [17,18,19]. On the other hand, due to their specific cargo, they have a great potential to serve as biomarkers for the diagnostic and/or prognosis of pathologies [20,21].

In the last years, it has been demonstrated that the MSCs from human patients with certain immune-mediated diseases are dysfunctional, leading to a better knowledge of these cells and their involvement in diseases [22,23,24,25]. However, this relation has not yet been described in cats.

The main objective of this study was to analyze the proteomic profile of fAd-MSC-derived exosomes from FCGS patients who tested positive for calicivirus infection, and to evaluate the existence of alterations in the exosomal cargo. These data will help us to better understand the etiopathogenesis of this disease.

## 2. Materials and Methods

All the used protocols received approval by the Ethics Committee of the Andalusian Center for Nanomedicine and Biotechnology (BIONAND, Málaga, Spain). The procedures affecting animals were performed by veterinarians after obtaining informed consent from the owners, which approved the use of biological samples from their cats for this study.

### 2.1. Selection Criteria

Ten European shorthair cats were included in this study (5 healthy controls and 5 calicivirus-positive FCGS patients). The inclusion criteria for the FCGS group were as follows: severe FCGS confirmed by a biopsy, with all molars extracted at least 6 months earlier and being refractory to immunomodulatory therapies, not showing any clinical improvement [26]. None of the selected cats were spayed or neutered.

The cats with FCGS were selected after being subjected to a clinical exploration and hematological and biochemical tests to discard any other existing pathologies. Hyperproteinemia, hyperglobulinemia, and neutrophilia were accepted, as they have been reported to be related to FCGS. The absence of dental roots was confirmed by dental radiographs [27]. The selected cats were tested positive for calicivirus by RT-PCR on oropharyngeal samples and negative for the feline immunodeficiency and leukemia viruses (SNAP Combo FeLV/FIV, IDEXX Laboratories, Westbrook, ME, USA) [26]. The severity of the disease was evaluated using the Stomatitis Disease Activity Index (SDAI) [28], selecting patients that scored a minimum of 20 points out of 30.

The healthy control cats did not have any known pathology. They were subjected to the same clinical and laboratory tests and were proved negative for the aforementioned viral infections.

No medication was given to the cats for at least ten days prior to the extraction of adipose tissue. Only analgesic drugs were allowed for the FCGS patients for palliative purposes.

### 2.2. Isolation and Expansion of fAd-MSCs

A subcutaneous adipose tissue sample (~5 g) was extracted from the inguinal area of each cat after sedation for other procedures not related to this study (clinical examination not involving traumatic procedures). The cats were sedated with ketamine (Imalgene, Merial, France; 10–20 mg/cat, IV), midazolam (Midazolan B. Braun, Melsungen, Germany; 0.1 mg/kg, IV), and butorphanol (Turbogesic, Zoetis, Madrid, Spain; 0.1 mg/kg IV). The obtained samples were maintained in Dulbecco’s modified Eagle medium (DMEM, Merck, Darmstadt, Germany) at 4 °C until taken to the laboratory on the same day.

Further, fAd-MSCs of each donor were isolated and characterized according to a protocol previously optimized within our group [15,29]. Briefly, adipose tissue was treated with type I collagenase (Merck, Darmstadt, Germany) and the isolated fractions were seeded in T-175 culture flasks containing DMEM supplemented with 10% fetal bovine serum (FBS), 2.5 mM L-glutamine, 100 U/mL penicillin, 100 µg/mL streptomycin, and 1.25 µg/mL fungizone (Merck, Darmstadt, Germany). The fAd-MSCs were cultured under standard conditions (37 °C, 5% CO_2_, 95% RH) and subcultured when reaching >80% confluency. All experiments were performed with cells at passage 2. The fAd-MSCs were characterized by flow cytometry, and their multipotential differentiation capacity into adipogenic, osteogenic and chondrogenic lineages, according to the criteria of the International Society of Cellular Therapy (ISCT), were evaluated using protocols previously published by our group [29,30].

### 2.3. Isolation of fAd-MSC-Derived Exosomes

The exosomes from each individual donor were processed separately. The isolation of the exosomes was conducted by ultracentrifugation and their characterization afterwards was conducted following a protocol previously used by our group [13,15]. Briefly, the cells of each donor were seeded at a density of 5.7 × 103 cells/cm^2^ in three T-175 flasks in DMEM supplemented with 10% fetal bovine serum (FBS), 2.5 mM L-glutamine, 100 U/mL penicillin, and 100 µg/mL streptomycin (Merck, Darmstadt, Germany). When 80% confluency was reached, the cells were washed with PBS and DMEM with 10% exosome-free FBS was added to the flasks. The exosome-free FBS was previously obtained by ultracentrifugation at 100,000× *g* for 60 min at 4 °C. The culture medium containing the fAd-MSC-derived exosomes was collected after 72 h under standard culture conditions. The cells remaining in the flasks were quantified and their overall viability determined by trypan blue staining and loading in a Neubauer chamber.

The culture medium was centrifuged at 13,000× *g* for 30 min at 4 °C to eliminate the cell debris and microvesicles. Afterwards, it was ultracentrifuged at 100,000× *g* for 90 min at 4 °C using a fixed-angle 70Ti rotor in an Optima LE-80K ultracentrifuge (Beckman Coulter, Pasadena, CA, USA). The sedimented exosomes were collected by resuspending them in PBS before repeating the ultracentrifugation step. Finally, the exosomes were resuspended in 100 µL of PBS, quantified using a BCA kit (Thermo Fisher Scientific, Waltham, MA, USA) and stored at −80 °C until needed. For the characterization of the exosomes, equal amounts coming from each donor were combined.

### 2.4. TEM Analysis

Aliquots of the isolated exosomes were adsorbed onto formvar/carbon-coated 200 mesh grids, negatively stained with 2% aqueous uranyl acetate and observed using a JEM-1400 transmission electron microscope (JEOL, Tokyo, Japan), operated at 80 kV following directions from the Transmission Electron Microscopy Unit from the Central Research Support Service (SCAI) of the University of Malaga.

### 2.5. Size Distribution and Exosomal Electronegativity

Size, distribution, homogeneity, and zeta potential of the exosome fractions (20 µL) that were previously diluted in 1 mL of distilled water were analyzed using a Malvern Zetasizer 2000 (Malvern Instruments, Worcestershire, UK). Each sample was analyzed in triplicate to obtain the size distribution of the exosome population.

### 2.6. Analysis of the Expression of Exosome Markers by Western Blot

The presence of exosome-specific biomarkers in the isolated fractions was evaluated by Western blot [31,32,33,34]. Then, 30 µg of exosomes were used for performing an SDS-PAGE followed by a semi-dry transfer to nitrocellulose. Rabbit monoclonal antibodies against the specific exosomal markers ALIX (Abcam, Cambridge, UK; 1:1000) and the tumor susceptibility gene 101 TSG101 (Abcam, Cambridge, UK; 1:1000), as well as against calnexin (Cell Signaling Technology, Danvers, MA, USA; 1:1000) and polyclonal β-actin (Abcam, Cambridge, UK; 1:2000), followed by incubation with peroxidase-conjugated polyclonal goat anti-rabbit IgG (Abcam, Cambridge, UK; 1:10,000). The marked proteins were visualized by incubation with ECL (Thermo Fisher Scientific, Waltham, MA, USA) using a ChemiDoc XRS+ system (BioRad, Hercules, CA, USA). The protein fraction of a lysed human adipose tissue-derived MSC sample was used as a positive control.

### 2.7. Exosome Protein Content Analysis by Ultra-High-Performance Liquid Chromatography High-Resolution Mass Spectrometry (UHPLC–HRMS)

The exosomal proteins were purified by a trichloroacetic acid protein precipitation-based procedure (clean-up kit; GE Healthcare, München, Germany) and the resulting protein pellets were recovered and quantified by a Bradford assay. Afterwards, gel-assisted proteolysis was carried out, entrapping the protein solution in a polyacrylamide gel matrix. Briefly, gel plugs containing the proteins were cut into small pieces and incubated with DTT to break disulfide bonds. Cysteine residues were carbamidomethylated with iodoacetamide and the proteins were digested with trypsin (Promega, Madison, WI, USA). The resulting peptides were then extracted from the gel, dried in a SpeedVacTM vacuum concentrator, and re-dissolved in 0.1% formic acid aqueous solution. Finally, the peptides were purified using a C18 ZipTip (Merck, Darmstadt, Germany) according to manufacturer’s instructions and brought to an identical concentration before being transferred to the injection vial.

Samples were injected into an Easy nLC 1200 UHPLC system coupled to a hybrid linear trap quadrupole Orbitrap Q-Exactive HF-X mass spectrometer (Thermo Fisher Scientific, Waltham, MA, USA). The samples were automatically loaded onto a trap column (Acclaim PepMap 100, 75 um × 2 cm, C18, 3 um, 100 A, Thermo Fisher Scientific, Waltham, MA, USA) at a flow rate of 20 uL/min and eluted through a 50 cm analytical column (PepMap RSLC C18, 2 um, 100 A, 75 um × 50 cm, Thermo Fisher Scientific, Waltham, MA, USA). The binary gradient mobile phase consisted of 0.1% formic acid in water (solvent A) and 0.1% formic acid in 80% acetonitrile (solvent B). Peptides were eluted from the analytical column with a 120 min gradient ranging from 2% to 20% solvent B, followed by a 30 min gradient from 20% to 35% solvent B and finally, to 95% solvent B for 15 min before re-equilibration at a constant flow rate of 300 nL/min.

Data acquisition was performed in the electrospray ionization positive mode. MS1 scans were acquired from *m*/*z* 300–1750 at a resolution of 120,000. The 20 most intense precursor ions were isolated within a 1.2 *m*/*z* window by a data-dependent acquisition method and fragmented to obtain the corresponding MS/MS spectra. The fragment ions were generated in a higher-energy collisional dissociation (HCD) cell and detected in an Orbitrap mass analyzer at a resolution of 30,000.

Acquired raw data were analyzed using the Proteome DiscovererTM 2.3 platform (Thermo Fisher Scientific, Waltham, MA, USA) with SEQUEST^®^ HT as search engine and *Felis catus* NCBI version 2017.10.30 (43.896 sequences) as database. Peptide spectral matches and consecutive protein assignments were validated using the Percolator^®^ algorithm [35] by imposing a strict cut-off of 1% false discovery rate (FDR). Results were filtered to contain only proteins with at least two unique peptide sequences.

Label-free quantitation was implemented using the Minora feature of Proteome DiscovererTM 2.3. Protein abundances were based on precursor intensities. Normalization was performed based on total peptide amount and abundance ratio p-values were calculated by ANOVA based on individual protein abundances.

Biological process, molecular function and cellular component enrichment analysis of the identified proteins was carried out via the international standardized gene function classification system of gene ontology (http://www.geneontology.org/- accessed on 12 February 2021) using the network-based annotation service of the Proteome DiscovererTM 2.3 application.

## 3. Results

### 3.1. Selection of Cats

Five FCGS-positive and five healthy European shorthair cats were selected for this study. Each group contained three males and two females, with an average weight of 3.4 ± 0.6 kg for the FCGS group and 3.8 ± 0.24 kg for the control group. The average ages were 6.4 ± 3.7 years and 4.2 ± 1.3 years, respectively, and the average SDAI score in the FCGS group was 22.4 ± 0.9 (Table 1).

### 3.2. Characterization of fAd-MSCs

The isolated fAd-MSCs of all the donors were homogeneous CD29+, CD44+, CD73+, CD90+, MHC-I+ (mesenchymal stem cell markers), CD34−, CD45−, and MHC-II- (hematopoietic markers) cell populations (data not shown). These fAd-MSCs differentiated to the three mesodermal lineages after appropriate induction. Adipogenic differentiation was evidenced by the presence of Oil Red O-positive fat drops; osteogenic differentiation was confirmed by alizarin red-positive calcium deposits; and chondrogenic differentiation was confirmed by the formation of micromasses exhibiting metachromasia when stained with toluidine blue (Figure S1).

### 3.3. Characterization of fAd-MSCs-Derived Exosomes

A total of 13.35 ± 4.04 µg exosomes/10^6^ fAd-MSCs were isolated from the FCGS-positive cats and 7.12 ± 6.3 µg exosomes/10^6^ fAd-MSCs from the healthy donors. The exosome fractions were analysed by TEM and dynamic light scattering (DLS). The exosomes that were isolated from the FCGS cats had a clearly lower average size and z-potential compared to the exosomes that were obtained from the healthy cats (Table 2).

**Figure 1:**
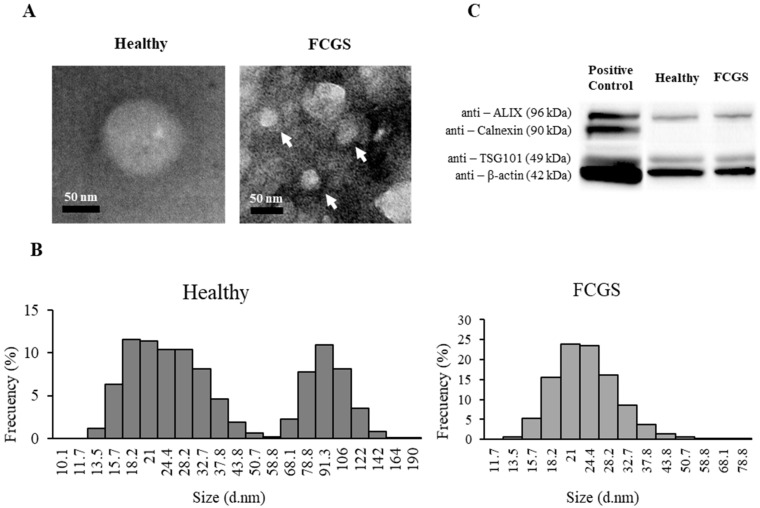
Characterization of the fAd-MSC-derived exosomes from FCGS-positive and healthy cats. (**A**) TEM images of exosomes in both groups. White arrows point to exosomes. Other structures may be precipitated salts present in the buffer. Scale bars: 50 µm. (**B**) Western blot analysis showing the presence of specific exosomal markers in both groups. (**C**) Exosomal size distribution, showing a loss of the exosome subpopulation with a greater diameter in FCGS cats.

The low polydispersity of the samples indicates a low aggregation of the exosomes. This was also observed by TEM visualization (Figure 1A). The size distribution, within the exosome sample from the healthy donors, ranged between 13 and 140 nm, with two clear subpopulations (13–50 nm and 68–140 nm). The larger subpopulation was, however, not present in the samples that were obtained from the FCGS-positive cats (Figure 1C).

The exosomes that were obtained from the fAd-MSCs of both the groups displayed specific exosomal markers, such as ALIX or TSG101, involved in exosome biogenesis and vesicular trafficking, and were negative for calnexin, which is an endoplasmic reticulum-related marker that is commonly used as a negative control for exosome identification (Figure 1B).

### 3.4. Comparison between the Protein Cargos of fAd-MSC-Derived Exosomes from FCGS and Healthy Cats

After performing the proteomic analysis, and according to the *Felis catus* database, a total of 508 proteins were identified in the exosomes from the FCGS-positive cats, while 506 proteins could be found in the exosomes from the healthy donors. The complete lists of proteins can be found in Table S1. We were able to identify eight proteins that were exclusively present in the exosomes from the FCGS group, and five proteins that were exclusively present in the exosomes from the healthy cats (Table 3 and Table S1).

### 3.5. Gene Ontology (GO) Analysis of the Identified Proteins

**Figure 2:**
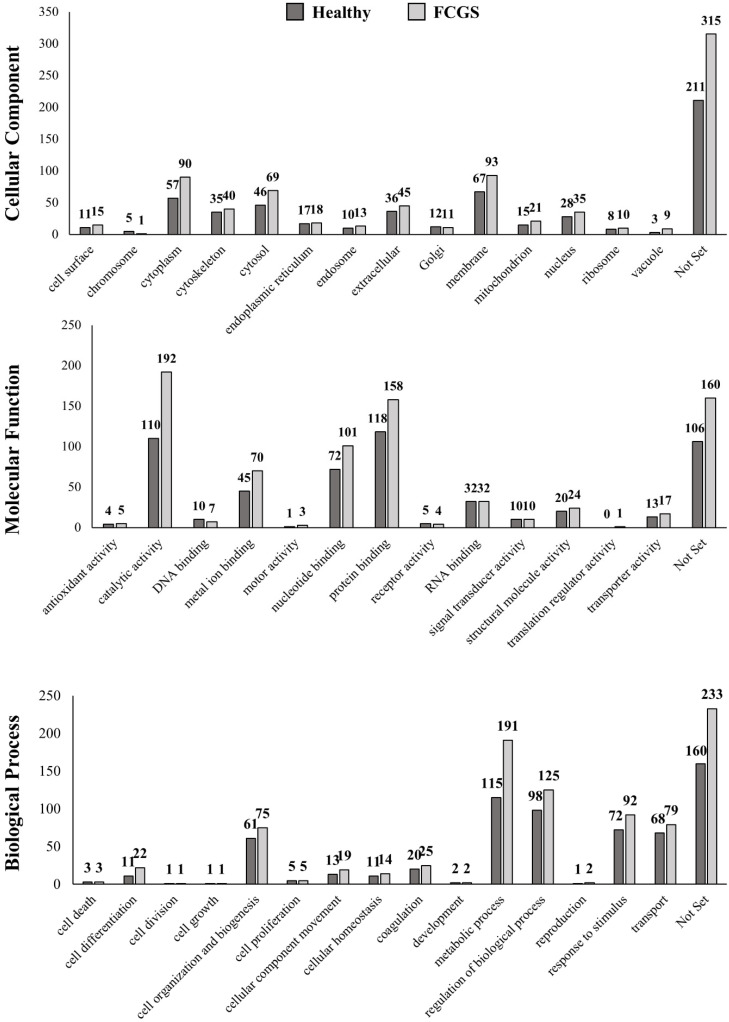
GO annotations related to proteins found in the fAd-MSC-derived exosomes. Subcellular localization, molecular function, and biological processes related to the identified proteins. A single protein can be included in different subgroups. “Not set” indicates that these proteins are not classified in any of the parameters described according to GO (more information is provided in Table S1).

Each identified protein was functionally annotated, based on the following GO parameters: cellular component, molecular function, or biological process (Figure 2). The most abundant proteins in both the groups were classified as membrane proteins, followed by proteins localized in the cytoplasm (referring to the entire content of a cell, excluding the plasmatic membrane and the nucleus, but including other subcellular structures), the cytosol (the part of the cytoplasm that does not contain organelles, but contains other particles, such as protein complexes), and extracellular region proteins. All these proteins were more abundant in the FCGS group than in the control group. In addition, the exosomes from the FCGS group were enriched with proteins that were involved in the metabolic process, regulation of the biological process, response to stimulus, transport, and cell organization and biogenesis. According to the molecular function of the identified proteins, the predominant protein fraction in the exosomes from the FCGS group was related to catalytic activity, protein binding, nucleotide binding, and metal ion binding, in this order. Contrarily, the exosomal proteins that were found in the healthy donors were predominantly involved in protein binding. Only among the groups related to DNA binding and receptor activity could more proteins be identified in the FCGS than in the control group. The complete list of the proteins is provided in Table S1.

### 3.6. Label-Free Quantification of the Exosome Proteomes

**Figure 3:**
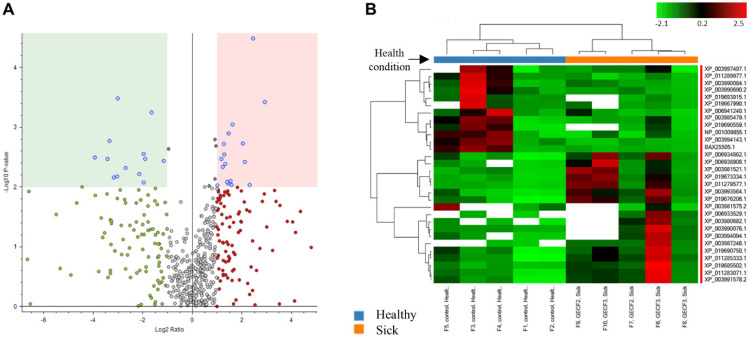
Deregulated proteins in FCGS patients. (**A**) Volcano plot representing the down-expressed and overexpressed protein abundance changes (green circles). Proteins were considered downregulated or upregulated if their fold-change values were <0.5 or >2, respectively, with a *p*-value < 0.01. (**B**) Heat map showing the protein levels exosomes isolated from FCGS cats (orange bar) versus healthy donors (blue bar).

When comparing the exosomal cargo between the two groups, significant upregulation of 17 and downregulation of 13 proteins were found in the FCGS group compared to the control group, with a *p* < 0.01 (Figure 3). These numbers were even higher (20 upregulated and 86 downregulated proteins) when a *p* < 0.05 was considered. The list with these specific proteins can be found in Table S2.

The upregulated proteins are mainly involved in metabolic processes (sorbitol dehydrogenase, uroporphyrinogen decarboxylase, alpha-1,3-mannosyl-glycoprotein, 2-beta-N-acetylglucosaminyltransferase, puromycin-sensitive aminopeptidase, neutral alpha-glucosidase AB, dipeptidyl peptidase, phosphoglucomutase-1), hepatocellular function and development (oncoprotein-induced transcript 3), immune response (complement C1s subcomponent), regulation of biological processes (tyrosine protein kinase receptor Tie-1, EMILIN-1, microtubule-associated protein RP/EB, transforming growth factor beta-1-induced transcript 1 protein, cullin-associated NEDD8-dissociated protein 1), gene expression (threonine-tRNA ligase), vesicular transport (general vesicular transport factor p115), and protein folding (heat shock 70 kDa).

The proteins that were, on the contrary, downregulated in the exosomes from the FCGS cats, were mainly involved in cell adhesion (annexins, cadherins, catenin, integrins), mitochondrial metabolism (NADH-cytochrome b5 reductase), cell surface receptor (CD63), membrane and cytoskeletal dynamics (protein S100-A11), metabolic processes (peroxiredoxin-1; UDP-glucose 6-dehydrogenase), regulation of biological processes (urokinase plasminogen activator surface receptor, myeloid-associated differentiation marker), proliferation (guanine nucleotide-binding protein G), and RNA binding (poly(rC)-binding protein 2).

## 4. Discussion

The use of MSCs is an interesting therapeutic alternative for a large number of immune-mediated pathologies, due to the ability of these cells to modulate the immune system [36,37,38]. It is known that the exosomes that are produced by these cells play a pivotal role in this ability, as they are related to cell-to-cell communication in both healthy and diseased conditions [32,39]. However, very little is still known about the role of exosomes and their possible alterations in immunocompromised pathologies, especially in veterinary patients. In this study, we report, for the first time, the isolation and characterization of exosomes obtained from the fAd-MSCs of calicivirus-positive patients with severe and refractory FCGS, and we demonstrate that these have an altered proteomic profile, which might eventually lead to a dysfunctionality. Adopting the infection by calicivirus, as an inclusion criterium, was due to the high impact of this viral infection on the cat population suffering from FCGS [2,3,4,40].

It has been estimated that around 50% of the fAd-MSCs might be infected with feline foamy virus [41], which seems to affect their proliferation after passage 3. Therefore, we isolated the exosomes from the fAd-MSCs in passage 2, which allowed us to obtain enough cells for the experiments, while avoiding unnecessary additional subculturing, which might have modified the properties of the cells [29,42].

The fAd-MSCs from the cats with FCGS produced notably more exosomes than the cells from the healthy donors (almost double the amount), as has been previously reported for other pathologies [43]. Furthermore, the exosomes from the diseased cats show a lower zeta potential. Behaving similarly to nanoparticles, the superficial charge of the exosomes is reflected on their zeta potential and this characteristic varies depending on the origin of the exosomes [44]. The zeta potential measures the charge stability and affects all the functional interactions between particles. So, a higher zeta potential leads to greater electrostatic repulsion forces between the particles, increasing their colloidal stability and reducing their chances of aggregating [45]. In this sense, it was recently described that there are dimer-forming exosome–exosome interactions that can modulate the signals that are provided by those exosomes in different biological processes, such as the immunoregulation of cancer, metastasis, or tissue regeneration [46]. Therefore, we believe that more studies on the exosomal interactions in veterinary patients with FCGS could yield very interesting data in the future.

The proteomic analysis of the exosome cargo, by high-resolution mass spectrometry, is a very useful tool for comparing the protein profiles in exosomes from different origins, and can lead to conclusions on their role in normal biological and physiopathological processes [47]. A total of 508 proteins were identified in the exosomes from the FCGS patients and 506 proteins could be found in those derived from the healthy animals. When quantifying the proteins that were shared by both the groups, we found several alterations in the proteomic profile of the exosomes from the diseased cats. On the one hand, eight proteins were only present in the exosomes that were derived from the fAd-MSCs isolated from the FCGS patients, while five proteins could only be found in the samples from the healthy cats. The exosomal proteins that were identified exclusively in diseased animals were related to the regulation of biological processes, such as tissue remodeling, inflammation, defense against infectious agents, and metabolism [48,49,50,51]. The proteins that were missing in the FCGS group were involved in the immune response, inflammation, chromatin structure modulation, transcription, replication, recombination, DNA repair, and gene silencing [52,53].

On the other hand, 17 proteins were significantly more represented in the exosomes from the FCGS group, mainly related to metabolic processes, immune responses, regulation of biological processes, and gene expression. Thirteen other proteins were downregulated and these were mainly involved in metabolic functions.

These differences confirmed that there are evident changes in the secretory profile of the fAd-MSCs, and the proteomic cargo of their exosomes, from cats with severe FCGS. The amount and variety of expressed proteins show that certain biological functions, related to tissue regeneration, metabolism, and immune responses, may be affected. These processes may be involved in the pathogenesis of FCGS and might help to understand what role the exosomes play in certain immune-mediated pathologies [54]. Furthermore, this represents a promising strategy for gaining information on the response to pathogens, identifying therapeutic targets and selecting specific biomarkers for different pathologies [55,56,57]. New studies will be needed to determine the possible role that the calicivirus or other concomitant viral infections might have in the proteome cargo of fAd-MSC-derived exosomes, or that of other origins in FCGS patients. In the current study, it was not possible to determine whether the altered proteomic profile of the fAd-MSC-derived exosomes from the FCGS patients was due to the gingivostomatitis, the calicivirus infection, or a combination of both.

Although complete teeth extraction usually leads to a high percentage of improvement in cats suffering from severe FCGS, around 30% of the patients are refractory to the existing treatments [8,28]. Therefore, new studies would be needed on patients in the early stages of the disease, when no teeth have been extracted yet, to compare to our results with severe and refractory cases. This could help in identifying biomarker proteins, to reduce the high percentage of cases that do not satisfactorily respond to treatments and assure the success of the exodontias.

Recently, the use of both autologous and allogenic MSCs has been proven to be useful for the treatment of this pathology [37,38]. Hence, taking our findings into account, a better knowledge of the secretory profile of MCSs is needed for interpreting the results of possible future therapies with autologous cells, in patients suffering from severe FCGS.

Currently, the use of exosomes, as therapeutic agents in different disease models, has been shown to be equally as safe and efficient as the direct use of MSCs [58,59]. Furthermore, thanks to their specific cargo, they have gained an interesting role as biomarkers for the diagnosis and/or prognosis in different pathologies, including cancer [21,60]. In any case, approaches based on the use of allogenic MSCs or their exosomes have a greater chance of being approved for commercialization by the authorities than those based on autologous cells, as the allogenic cells allow for a better control of their efficacy and have important logistical advantages.

The number of animals studied, their age differences, and the limited amount of information available in the cat databases, are the main limitations of our study. Furthermore, since the exosomes from each donor could not be analyzed separately, possible inter-individual heterogeneity might have been lost. However, our results might set a base for the future identification of robust groups of proteins present in the fAd-MSC-derived exosomes from healthy and diseased animals. This would allow us to perform more studies on the use of exosomes and their cargo, for either diagnostic or therapeutic purposes.

Finally, since the domestic cat suffers with FCGS naturally and in environmental conditions that are similar to those in which humans live, it might be a suitable preclinical model for transferring these results to some human pathologies, such as oral lichen planus, stomatitis, pemphigus, or pemphigoid [61].

## 5. Conclusions

In this study, the alteration of the proteomic profile of fAd-MSC-derived exosomes, from severe, calicivirus-positive FCGS patients, is presented for the first time. Using bioinformatic tools, we have demonstrated that the altered proteomic profile in the exosomes from diseased patients affects the proteins related to several biological functions, such as tissue remodeling, metabolism, and immune responses. The exosomes do not only experience changes in their cargo, but are also produced in larger quantities. Possibly, the alterations in the exosomes in these patients will change their ability to participate in cell communication between the Ad-MSCs and their niche, which is affected by this pathology.

## Figures and Tables

**Table 1:** Information about the animals selected for the FCGS and the control group. The sex, age, weight and SDAI score are presented. None of the selected cats were spayed or neutered.

Group	Sample Number	Sex	Age (years)	Weight (Kg)	SDAI ^1^ Score
FCGS ^2^	1	Male	9	3.4	22
	2	Male	11.5	3.3	23
	3	Male	2.5	2.9	23
	4	Female	5	4.5	21
	5	Female	4	3.1	23
	**Mean**		6.4	3.4	22.4
	**SD** ^3^		3.7	0.6	0.9
Healthy	6	Male	6	3.8	N/A ^4^
	7	Male	4	4	
	8	Male	3	4.1	
	9	Female	5	3.5	
	10	Female	3	3.7	
	**Mean**		4.2	3.8	
	**SD**		1.3	0.24	

^1^ SDAI: Stomatitis Disease Activity Index. ^2^ FCGS: feline chronic gingivostomatitis. ^3^ SD: standard deviation. ^4^ Not applicable.

**Table 2:** Characterization of the isolated exosome samples.

Group	Quantification (µg Exosomes/10^6^ Cells)	Size Range (nm)	Electronegativity (Zeta Potential)	Polydispersity Index (PDI)
FCGS	13.35 ± 4.04	15–50	−13.4 ± 3.82 mV	0.277 ± 0.09
Healthy	7.12 ± 6.3	15–140	−22.7 ± 0.71 mV	0.311 ± 0.03

**Table 3:** Proteins exclusive to the exosomes of a certain group. A total of eight proteins were only identified in the exosomal proteome within the FCGS group and five proteins were exclusively found in the exosomes of the healthy donors.

Group	Accession	Protein Name	Number of Peptides	Coverage (%)
FCGS	XP_006928390.1	Tubulin beta-4A chain	18	70
	ACO24947.1	Pancreatic amylase, partial	3	7
	XP_003984122.1	Pleckstrin	2	9
	XP_019676911.1	Protein Niban	2	3
	XP_003993984.1	Ras-related protein Rab-4A isoform X1	2	11
	XP_011280274.1	Dihydropteridine reductase	2	14
	XP_019683254.1	Latent-transforming growth factor beta-binding protein 1 isoform X1	2	3
	XP_019670812.1	Endothelial lipase isoform X1	2	7
Healthy	XP_019687676.1	Poly [ADP-ribose] polymerase 6 isoform X11	30	61
	XP_003985767.1	Histone H1.4	6	23
	XP_003985772.1	Histone H1.2	6	24
	XP_003984106.1	Ras-related protein Rab-1A	7	36
	XP_003985733.1	Histone H1.5	3	18

## Data Availability

The data presented in this study are available in this published article (and its Supplementary Materials).

## Supplementary Materials

The following are available online at https://www.mdpi.com/article/10.3390/ani11082466/s1, Figure S1: assessment of adipogenic, osteogenic and chondrogenic differentiation; Table S1: list of proteins present in the FCGS group and healthy donors, and proteins exclusive to the exosomes of a certain group; Table S2: complete list of upregulated and downregulated proteins with a value of *p* < 0.01.
