# Supplementary material for: Altered Proteomic Profile of Adipose Tissue-Derived Mesenchymal Stem Cell Exosomes from Cats with Severe Chronic Gingivostomatitis
Source: Animals (Basel). 2021 Aug 23;11(8):2466. doi: 10.3390/ani11082466 (PMC8388770; doi:10.3390/ani11082466)
Supplement: Supplementary file 1 [file animals-11-02466-s001.zip › Supplementary table S2.pdf]

Supplementary Table S2. Proteins up- and down-regulated ($p < 0.01$) in the exosomes from the FCGS patients.

Number		Accession	Description	Peptides	Coverage [%]	Abundance Ratio: Sick / Healthy	Abundance Ratio P-Value: Sick / Healthy
UP-REGULATED PROTEINS	1	XP_003987246.1	Sorbitol dehydrogenase	3	11	**7,653**	0,0004
	2	XP_003990076.1	Uroporphyrinogen decarboxylase isoform X1	4	9	**5,480**	0,0000
	3	XP_003980682.1	Alpha-1,3-mannosyl-glycoprotein 2-beta-N-acetylglucosaminyltransferase	2	6	**4,976**	0,0093
	4	XP_003994094.1	Oncoprotein-induced transcript 3 protein isoform X1	3	6	**4,375**	0,0038
	5	XP_006933529.1	Complement C1s subcomponent	3	5	**4,108**	0,0019
	6	XP_019673334.1	Puromycin-sensitive aminopeptidase isoform X1	9	11	**3,090**	0,0009
	7	XP_003993564.1	Neutral alpha-glucosidase AB isoform X1	9	13	**3,018**	0,0092
	8	XP_011283071.1	Tyrosine-protein kinase receptor Tie-1	6	7	**2,917**	0,0080
	9	XP_011279577.1	EMILIN-1	16	25	**2,797**	0,0013
	10	XP_006938908.1	Microtubule-associated protein RP/EB family member 2 isoform X3	8	28	**2,746**	0,0087
	11	XP_003981521.1	Threonine-tRNA ligase, cytoplasmic	6	10	**2,652**	0,0083
	12	XP_019685502.1	General vesicular transport factor p115	8	10	**2,537**	0,0042
	13	XP_006934862.1	Phosphoglucomutase-1 isoform X3	14	28	**2,450**	0,0029
	14	XP_003991578.2	Heat shock 70 kDa protein 13	5	12	**2,433**	0,0019
	15	XP_011285333.1	Dipeptidyl peptidase 3 isoform X1	2	3	**2,345**	0,0046
	16	XP_019676206.1	Transforming growth factor beta-1-induced transcript 1 protein isoform X1	2	4	**2,276**	0,0034
	17	XP_019690750.1	Cullin-associated NEDD8-dissociated protein 1 isoform X2	10	10	**2,023**	0,0095
DOWN-REGULATED PROTEINS	1	XP_019690559.1	Poly(rC)-binding protein 2 isoform X1	2	5	**0,452**	0,0037
	2	XP_003985479.1	UDP-glucose 6-dehydrogenase	12	35	**0,320**	0,0006
	3	XP_003990084.1	Peroxiredoxin-1	9	42	**0,269**	0,0034
	4	XP_003997497.1	Myeloid-associated differentiation marker	2	10	**0,258**	0,0084
	5	NP_001009855.1	CD63 antigen	2	8	**0,257**	0,0028
	6	XP_011289977.1	Guanine nucleotide-binding protein G(i) subunit alpha-2	9	33	**0,229**	0,0061
	7	XP_006941240.1	Urokinase plasminogen activator surface receptor isoform X1	3	10	**0,156**	0,0048
	8	BAX25505.1	NADH-cytochrome b5 reductase 3	2	10	**0,125**	0,0003
	9	XP_003990690.2	Protein S100-A11	4	42	**0,124**	0,0068
	10	XP_003981575.2	Cadherin-6 isoform X1	2	3	**0,112**	0,0069
	11	XP_003994143.1	Annexin A11	5	10	**0,098**	0,0017
	12	XP_019667990.1	Catenin delta-1 isoform X1	2	3	**0,094**	0,0034
	13	XP_019693915.1	Integrin alpha-V	2	2	**0,066**	0,0033
