# Supplementary figures and images for: Altered Proteomic Profile of Adipose Tissue-Derived Mesenchymal Stem Cell Exosomes from Cats with Severe Chronic Gingivostomatitis
Source: Animals (Basel). 2021 Aug 23;11(8):2466. doi: 10.3390/ani11082466 (PMC8388770; doi:10.3390/ani11082466)

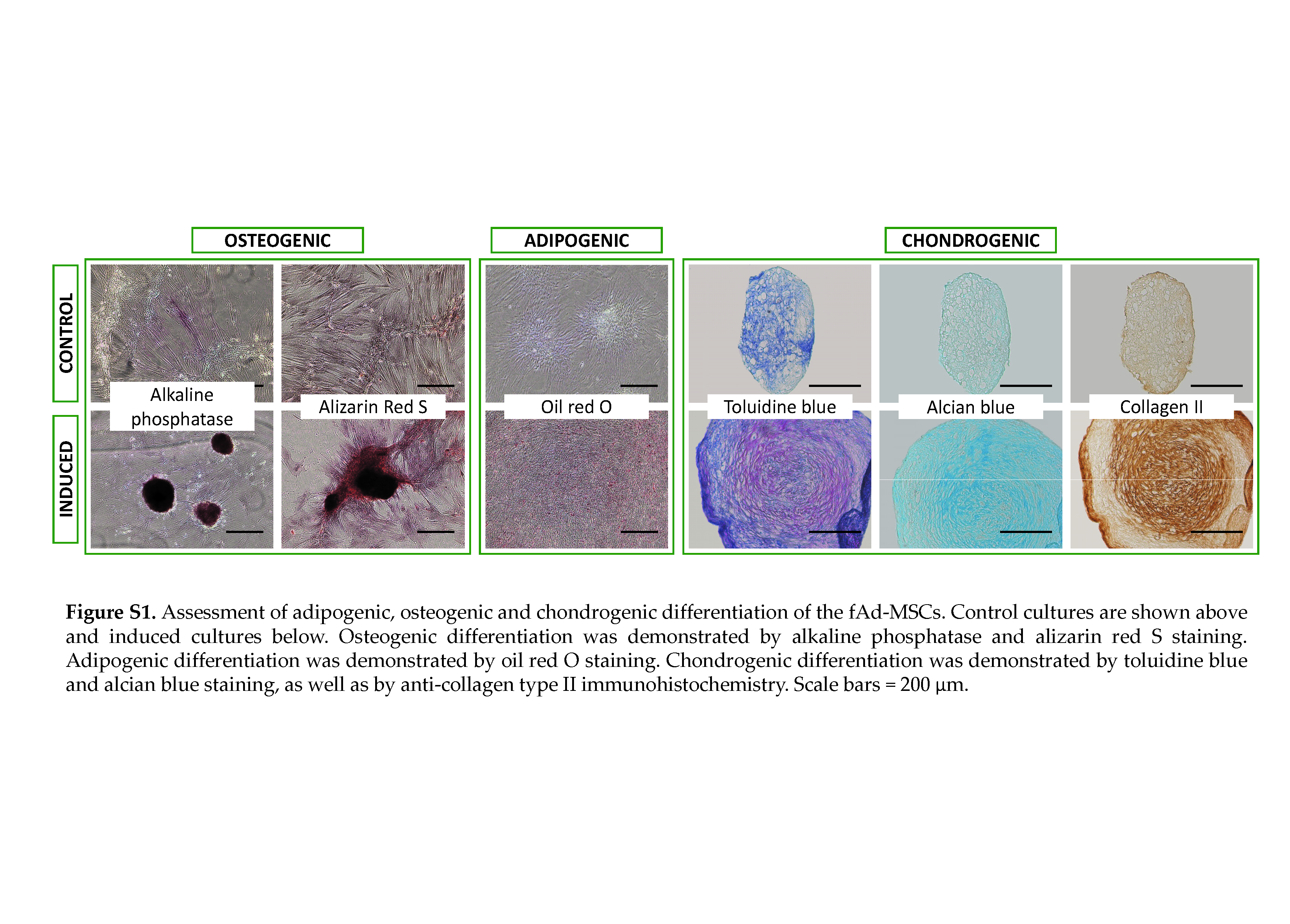

Supplement: Supplementary file 1 [file animals-11-02466-s001.zip › Figure S1_Assessment of adipogenic, osteogenic anf chondrogenic differentiation.tiff]
